# Situational awareness and situational heroism: a close reading of testimonies from the London Bridge terror attack 2019

**DOI:** 10.1080/21624887.2024.2388319

**Published:** 2024-08-05

**Authors:** Katharina Karcher

**Affiliations:** Department of Modern Languages, University of Birmingham, Birmingham, UK

**Keywords:** Situational awareness, terrorism, London Bridge, situational heroism, testimony, affect

## Abstract

Based on an-in depth analysis of two testimonies, this article explores how ordinary people can become ‘situational heroes’. On 29 November 2019, Darryn Frost and Steve Gallant were caught up in a terrorist attack in central London. In that situation, Frost and Gallant demonstrated a high degree of situational awareness and took bold and swift action to stop the attacker. Using the literary method of close reading, I analyse their testimonies with a particular eye towards two questions: how did the two men perceive the dangerous situation? Can and should they be seen as heroes? As a convicted murderer and long-serving prisoner, Gallant was certainly no hero in the conventional sense. Yet, in 2023 he received The Queen’s Gallantry Medal for ‘for acts of exemplary bravery’ alongside with Frost and a few others. This, I argue, is symptomatic of a broader shift towards situational awareness and towards what I call situational heroism. As it is less about *being* a hero and more about*acting heroically* in particular situations, situational heroism is less elitist and more inclusive than many historical forms of heroism. However, it also raises new ethical questions.

## Introduction

On 29 November 2019, Darryn Frost and Steve Gallant were caught up in a terror attack. On that day, the two men were attending an event at Fishmongers Hall bringing together former and current prisoners, educators, and volunteers involved in the pioneering ‘Learning Together Prison’ programme. Led by criminologists at the University of Cambridge, Learning together taught students alongside prisoners. Steve Gallant was one of the prisoners who had taken part in the programme. For that reason, he was given permission to attend the Learning Together event on 29 November 2019 accompanied by a prison officer. In the early afternoon, the event took a dramatic turn when Usman Khan began to stab people at Fishmongers’ Hall. Like Gallant, Khan was a former participant in the Learning Together Programme. Khan killed two people and injured several others before a small group of people including Gallant and Ministry of Justice employee Darryn Frost confronted him.

In 2023, Gallant and Frost received The Queen’s Gallantry Medal for their heroic efforts to stop Khan. Porter Łukasz Koczocik and ex-prisoner John Crilly were honoured with the same award because they helped confront the attacker. Prison officer Adam Roberts was commended for bravery for providing first aid to a stabbing victim at Fishmongers’ Hall. When Chancellor of the Duchy of Lancaster Oliver Dowden presented the men with their awards, he thanked them and highlighted the social importance of their bravery: ‘We all hope we’d react with courage in the face of danger. These people have lived through that test, and responded in the most admirable way’ (Cabinet Office 2023).

Frost, who was filmed by a member of the public using an ornamental narwhal tusk to keep Khan at bay, became quickly known as ‘Narwhal tusk’ hero. As this article shows, Gallant and other former or serving prisoners involved in stopping Khan were not seen as heroes in the same way as Frost. Yet, they were able to ‘shift into hero-mode’ (Krasmann and Hentschel 2019, 190) in the face of extreme danger.Frost, Gallant, Koczocik and Crilly were ordinary people who happened to be caught up in the same violent event. What distinguished them from many others in the room was a high degree of situational awareness: they perceived an affective change in the atmosphere in the room, assessed what they could hear and see, and made the brave decision to take bold and swift action.

Mica R. Endsley (1995, 60) famously defined situational awareness as ‘a person’s state of knowledge about a dynamic environment’. According to Endsley, situational awareness has three key components: first, perception of relevant elements in the environment; second, comprehension of the current situation; and third a projection of future status(es). Situational awareness is thus a complex process entailing ‘sensing, perceiving, reading, assessing, guessing or conjecturing a situation’ (Krasmann and Hentschel 2019, 186). Recent campaigns by theUK’s National Counterterrorism Security Office have aimed at promoting situational awareness among the population, e.g. by advising citizens to ‘stay alert’ and ‘trust their instincts’ (Protect UK 2023). In this context, heroism has an important role to play.

Like many other nations, Britain has long celebrated the heroic actions of individuals. It is no coincidence that most of these individuals happened to be men. ‘With the occasional, troublesome exception ofa Queen Bess, a Florence Nightingale or a Margaret Thatcher, the nationalepic has been predominantly a man’s story, and masculine prowess the dominant expression of national character (Dawson 1994, 13). Some hero figures were subversives, as the famous example of Robin Hood demonstrates who took from the rich to give to the poor. However, most of the nation’s‘great men’ (Dawson 1994) were soldiers, sailors, engineers, or politicians and enjoyed high social status.Frost and Gallant may be no heroes in the traditional sense, but their exceptionally brave actions on 29 November 2019 had a moral impact and an ‘appellative force’. According to Ulrich Bröckling (2020, 16), it is this very ability that characterises heroism in post-heroic times. In today’s world, ‘Homeric, Virgilian, Romantic and Wagnerian conceptions of heroism, conditioning representations of war, have lost theirglamour’ (Calder 2004, x). It is important to stress that this does not mark the end of heroism as an important social and political phenomenon. Rather, it signals a shift in public debates about heroism in post-heroic times: from the heroic subject to heroic action and from *being* a hero to *acting* heroically in a particular situation. In a nutshell, heroism has become situational.

The London Bridge attack 2019 received a great deal of media attention and became the subject of several books and a documentary film.^1^ However, it has not yet been analysed through the lens of situational awareness and heroism. How have the individuals involved perceived the affective atmosphere in the room? What made them realise that the situation was dangerous? And, what form of heroism emerged in this situation? To provide answers to these questions, I will offer a close reading of Steve Gallant’s autobiographical book *The Road to London Bridge: How I went from a life of violence to stopping the terror attack Fishmongers’ Hall* and two interviews that I carried out with Darryn Frost. Both testimonies provide fascinating insights into the affective force of violent situations. As we shall see, situational heroism requires a careful assessment of changes in affective atmospheres.

## Analysing affective atmospheres and situational awareness: from close reading to non-representational approaches (and back)

In the last two decades, affect has become an important concept in the Humanities and Social Sciences. Building on the work of a range of thinkers including Massumi (2002, 2005), Thrift (2004, 2007), and Anderson (2009, 2010, 2014), scholars in human geography and related fields have begun to document and analyse the affective experience of urban environments. Affect is difficult to define and even more difficult to measure. Phillip Vannini conceptualises affect as ‘a pull and a push, an intensity of feeling, a sensation, a passion, an atmosphere, an urge, a mood, a drive – all of the above and none of the above in particular’ (2015, 8–9). Vannini (2015) rightly stresses that there is no unique method to study affect. Yet, he is among a growing number of scholars in the Arts and Humanities insisting that a primary or exclusive focus on ‘human language’ (Thrift 2004, 59; see also Barad 2003) fails to do justice to the dynamic nature of affect.

Non-representational approaches to affect reject the focus on language and textual analysis associated with the Linguistic turn in the Humanities and Social Sciences. They tend to focus on ‘the unsaid and the barely sayable’ (Vannini 2015, 9). I agree that it is vital that researchers ‘pay greater attention to the ways in which they frame their research and its results, to what was *not* clearly stated, to shared experiences and the affects, feelings, and emotions they elevate on-the-ground’ (Shilon 2023, 4). However, this should not mean dismissing language (which is not really an option anyway). As I hope to show by building on the work of Paul Harrison (2010) and others, an in-depth analysis of testimonies in written and spoken form can play a vital role in this process.

Research on affective atmospheres and urban terrorism has never shunned language. Sara Fregonese and Sunčana Laketa, for example, combine ethnographic observations in Brussels and Paris with ‘scoping conversations’ with individuals and ‘fleeting conversations’ around key sites ‘to explore how (counter)terrorism feels’ in these two cities (Fregonese and Laketa 2022, 2). They conclude that the dynamic relations between human and non-human elements, built infrastructures, technologies, and crowds create ‘affective atmospheres’ that individuals and communities experience in different ways. *How* language in its many forms can inform research on affective atmospheres, however, remains underexplored. The pioneering studies of Elaine Scarry (1985) and Sara Ahmed (2004) have offered critical insights into the complex relationship between language, politics, and affect. In this tradition, this article makes the case for a deeper engagement with language in research on affective atmospheres and urban terrorism.

This article seeks to show that a *close reading* of testimonies by survivors of terrorist attacks can offer critical insights into the ways in which people sense affective atmospheres and experience situational awareness. Narrating and re-narrating an experience enables individuals to contextualise ‘an initially vague affect’ and to understand its continued emotional impact (Habermas 2018, 205). Testimonies are embodied, dynamic, and relational. According to Sara Jones (2019, 260), a testimony can be understood as ‘a communicative act in a given cultural context in which a witness gives an account of something he or she has directly experienced for the benefit of an audience that has not’. This can take many forms, including autobiographical texts, oral testimonies, film, and theatre. It is important to stress that testimonies do not necessarily represent past events in a way that is universally accepted as ‘authentic’ or ‘true’. Rather, they constitute situated and relational accounts of embodied processes of sensing and sense making and need to be analysed as such.

Close reading is a well-established method in Literary Studies, which gained prominence in the Anglo-American world in the 1940s (Lentricchia and DuBois 2003). Rather than focusing on authors and their intentions, close reading offers a detailed analysis of what they do *as texts* and how they work. Such a reading goes necessarily beyond language in the narrow sense. While testimonies can and should be read as texts, their affective power, ‘performative force’ (Krämer and Weigel 2017), and political significance are irreducible to language. According to Çaylı (2021, 485), assessing the political significance of testimonies ‘requires asking who is allowed to produce this knowledge in ways they see fit – in other words, who is granted epistemic agency’. If a close reading ignores the performative nature and political importance of testimonies, it will inevitably fail to do justice to this genre (Eaglestone 2002). And yet, as research on Holocaust testimony illustrates, it can be immensely productive to read testimonies as texts – especially if we pay careful attention to silences, interruptions, ambivalences, and contradictions.^2^ According to Harrison (2010, 163), these moments in testimonies demand that ‘we – as addressees – listen and read again, not only to try and understand better, more accurately and exactly, but also to try to hear and read our inability to hear and read, and so begin to outline our systems of thought and trace their hidden sources’.

The first testimony analysed in this article is a transcript of a two-part interview with Darryn Frost that I conducted via zoom in 2020 and 2023.^3^ Originally from South Africa, Frost works in communications at the Ministry of Justice and attended the Learning Together event on 29 November 2019 in a professional capacity. He became widely known as the ‘Narwhal Tusk hero’, because he was quickly identified as one of the key actors in the group who tried to stop Usman Khan (Karcher 2020). As Bröckling (2020) highlights, heroism requires heroic actions that are documented, shared, and praised. In Frost’s case, this happened through mobile footage showing him in action. The second testimony is Steve Gallant’s autobiography *The Road to London Bridge: How I went from a life of violence to stopping the terror attack Fishmongers’ Hall*, first published in October 2023. Gallant grew up in Hull, England. In 2005, he received a life sentence with a minimum of 17 years for killing father of two Barrie Jackson. Gallant was granted permission to attend the event at Fishmongers’ Hall with prison officer Adam Roberts because he had taken part in a range of Learning Together initiatives. Steve Gallant and Darryn Frost did not know each other when they joined forces to stop Usman Khan’s attack. From that moment on, their lives and testimonies were intertwined.

There are some important differences between Frost’s and Gallant’s testimony when it comes timing and delivery. Frost began to share memoriesof the attack with the public significantly earlier than other survivors.Roughly three weeks after the attack, he gave a first interview to the PA news agency. Over the next six months, Frost discussed his experience calmly and confidently on national TV and radio programmes (which is why I felt confident to approach him so soon after the traumatic experience). The research interview in July 2020 provided a more intimate setting than live TV interviews, and Frost had the opportunity to review the transcript. As a convicted murderer who was serving a prison sentence, Steven Gallant was not granted the same epistemic agency as Frost. Initially, prison authorities prevented Gallant from sharing his memories of the day with the public. His autobiography was published almost four years after the attack and more than two years after his release from prison.

Using NVivo, I have identified what I call sensory markers in Gallant’s autobiography and the transcripts of my interviews with Frost. Such sensory markers include verbs (e.g. ‘hear’, ‘see’, ‘feel’, and ‘shout’), adjectives (e.g. ‘crystal-clear’, ‘high-pitched’, and ‘bright’), and nouns (e.g. ‘screams’, ‘cold sensation’, ‘image’, ‘vision’). I have also highlighted words and expressions that the two men used to describe what they felt during and after the attack. Examples include: ‘Instinct took over’ (Gallant 2023, 253) and ‘I was probably wired’ (interview with Frost 2023). Finally, I have identified passages in which Gallant and Frost discuss the meaning of the ‘hero’ label. Following Harrison, I have read the two testimonies with an eye to silences, doubts, contradictions, and other moments hinting at the impossibility testimony (Harrison 2010, 163). Rather than interpreting such moments in the text as problems or failures, I read them as vital gestures towards constellations, dynamics, and processes that escape or exceed language.

## Sensing the situation

Jarrett Zigon (2015, 503) rightly emphasises that ‘a situation is both a singularity of which one has become a part, and a multiplicity that preexists one’s participation in it and, as already having been, exceeds this localized instance of it’. This openness distinguishes the situation from the event. According to Massumi (2011, 3), the event is ‘singular’ and ‘has an arc that carries it through its phases to a culmination all its own: a dynamic unity no other event can have in just this way’. Approaching the London Bridge attack 2019 as an event makes it possible to identify a clear beginning and end. The event was short: it began with Usman Khan’s attack on Jack Merritt in the toilets at Fishmongers’ Hall just before 2 pm and ended when he was fatally shot by police on London Bridge around 15 minutes later. The temporal dimension and boundaries of the situation, however, are far less clear.

Like most participants in the Learning Together event, Gallant and Frost had returned to their seats in the banqueting hall when Khan began to stab people outside. This meant that the people in the banqueting hall could hear but not see the attack. Gallant (2023, 252) describes how the atmosphere in the room changed in response to the sound outside:

Almost immediately after everyone had settled back down, a woman’s high-pitched screams cut through the atmosphere. They were coming from downstairs. A guest made a joke, to nervous laughter. But the screams continued. The mood changed in the room. It sounded like it could be something serious.

What Gallant describes here can be understood as an atmospheric change. Anderson and Ash (2015, 46) define atmospheric change as ‘as a matter of affects meeting one another in ways that produce (or fail to produce) new relations’ between human and non-human entities within a particular atmosphere. Atmospheres co-exist and intersect in complex ways, and they can change in response to internal and/or external dynamics. If the affective quality of an atmosphere changes as result of an encounter with another atmosphere, this is marked by a ‘tipping point’ (Anderson and Ash 2015, 47). Gallant’s testimony indicates that the sound of ‘high-pitched screams’ did not crush the joyful atmosphere in the room – at least not initially. In fact, someone tried to defuse the growing sense of anxiety with a joke. Yet, the screaming continued, and ‘confusion and concern spread like a wildfire around the room’ (Gallant 2023, 2). As highlighted in the previous section, testimonies should not be mistaken for accurate representations of historical events. They are relational and evolve over time. By suggesting that the screams were cutting through the atmosphere in the room, Gallant’s narrative evokes a knife at a point when he and others in the room did not know that Khan was stabbing people with one outside. So, when was the tipping point reached? It is here, where there are interesting differences between Gallant’s testimony and Frost’s. This illustrates that while atmospheres are shared, individuals sense them and make sense of them in different ways.

During his prison sentence, Gallant had witnessed numerous fights and violent attacks. In this environment, he had to rely strongly and sometimes exclusively on his hearing to assess what was happening around him. He learned through experience that ‘there’s a very distinctive sound’ when a fight breaks out on a prison wing: ‘officers running, the jangle of keys’ (Gallant 2023, 76). For Gallant, hearing is more than a sense: it is also an essential tool for making sense. Indeed, many of the sensory markers used by Gallant to describe the emerging situation relate to sound. Interestingly, this emphasis on sound runs through his testimony like a red thread. For example, Gallant includes a detailed description of how he and others shouted to warn unsuspecting pedestrians on London Bridge. And, in his description of the fight with Khan he uses words that give his readers a strong sense of what he heard. Examples include the words ‘Crunch!’ (254) (describing the sound of a narwhal tusk snapping on Usman Khan’s shoulder) and ‘Crack!’ (4) (describing what Gallant heard when he punched Khan in the jaw).

Darryn Frost’s testimony, by contrast, is dominated by visual sensory markers. When I interviewed him in 2020, he described his memories of the attack as ‘crystal clear’ and like ‘an HD … showing’. His testimony included detailed descriptions of objects and ornamental structures in the building. He appeared to remember almost everything in the building, ranging from little ‘dimly lit’ passageways to ‘the grand staircase’. During the interview, he used a range of gestures to explain what the environment looked and felt like (e.g. by making a spiral shape with his hands to describe what the double spiral staircase looked like). Interestingly, Frost rarely mentioned sound. In 2020, he told me that his ‘senses were heightened visually, but audibly everything else shut off, except for if it was pertinent to exactly what was happening’. In 2023, he added that he ‘never even realised the alarms were going off’.

It is notable that in Frost’s testimony there is no mention of the ‘high-pitched’ screams (which Gallant and other survivors of the attack described as the trigger for the atmospheric change). Of course, this does not mean that Frost did not hear the screams. Nor should we conclude from this that he is necessarily unable to remember hearing them. In fact, Frost gave an interview one month after the attack during which he reported that he and a few others sprang into action when they ‘heard the noise from the floor below’ (Givetash 2019). However, Frost’s testimony does not mark the screams as a tipping point. Rather it suggests that Frost understood that the situation was ‘really dangerous’ when he *saw* what was happening outside shortly after. When speaking to me in 2023, he explained that he looked over the banister and ‘saw Saskia [Jones] falling’. And, he saw another victim at the bottom of the stairs. Was his decision to investigate what was happening outside a conscious choice? The testimony leaves this and other questions around the emerging situation unanswered. This is not surprising, because it can be hard for survivors ‘to differentiate between intuition, just before, and a very rapid assessment of an emerging situation, just after, and an attempt to make sense of the random cruelty of disasters’ (Easthope 2022, 181).

When they looked down the stairs, Gallant and Frost were confronted with a horrific scene. Gallant describes the situation as chaotic: Adam Roberts was providing first aid to Saskia Jones, and another woman lay ‘in a pool of her own blood’. ‘People were hiding or running scared’, and screams ‘echoed around the illustrious surroundings’ (Gallant 2023, 2). Whilst signalling that he has detailed memories of the horrific scene, Frost was careful to avoid graphic descriptions of the victims. He told me that he ‘saw some of the injured people … uh, severely injured people’. The short hesitation and pause here is important. It signals an absence. It told me that Frost had seen a lot that remained unsaid. In the same interview, Frost had described the narwhal tusk and other objects in great detail. His decision to not talk about the injured and killed in the same detail can be understood as a sign of respect for the victims, but it is also a powerful gesture towards the traumatic impact of this scene. The affective force of the situation is irreducible to language. Of course, he could have tried to describe to me what he saw in the interview. Would I have been able to hear him and imagine the horror he saw and felt? Interestingly, Frost highlighted that there were times when he questioned the reliability of his own memory in relation to this horrific scene.^4^

Even without seeing the attacker at the bottom of the stairs, Frost felt that he had seen *enough* to conclude that he found himself in an extremely dangerous situation. He noted: ‘I hadn’t seen Khan, I didn’t know what was going to meet me down there, I just knew it was going to be horrible’. At this point, Frost had to assess a constantly evolving situation based on a range of known and unknown factors. Earlier on the day, he had taken a video of this very part of the building on his mobile phone to show his girlfriend the beauty of Fishmongers’ Hall. Now, he used this visual knowledge to assess what to do next. ‘I immediately knew that there was no weapon or anything to defend myself with down there, because I’d surveyed the place. Inadvertently, I’d surveyed it in quite [short pause] fine detail’. Frost took a narwhal tusk from the wall in a different part of the building and returned with this makeshift weapon. When he saw Gallant inside the foyer without any weapon, Frost gave him the tusk and ran back to get a second one.

Rather than prioritising their own safety and hiding, Gallant and Frost decided to confront Usman Khan. By taking on an attacker armed with two large knives who threatened to detonate a bomb (that later turned out to be fake), the two men took great risks. This counts particularly for Gallant, who was acting against the explicit instruction from his prison officer to stay put. A key difference between Gallant’s and Frost’s testimony is how their actions in that moment are contextualised.Gallant places great emphasis on the fact that he had made the conscious decision to renounce violence after killing someone in a fight in 2005. He also stresses that he had remained committed to this decision until he felt compelled to use force to protect others on 29 November 2019. Frost, by contrast, has no history of engaging in violent crime. He did not face the same pressure to explain and justify his decision to use force on London Bridge.

Rather than following a clear plan, Frost and Gallant reacted in a dynamic way to the movements of objects and people in their environment. Supported by a few others, they used narwhal tusks, a chair, and a fire extinguisher as makeshift weapons to keep Khan at bay. Whilst using violent means to achieve their aim, they avoided any excessive force (e.g. by stopping people from hitting and kicking Khan when he was defenceless) and remained focused on the task of protecting people from harm.This, too, was seen as heroic. As Bröckling (2020, 32) highlights, heroes are expected to strike a difficult balance: they need to be ready and willing to use violence whilst carefully avoiding the use of excessive force. This also applies to situational heroism. In many ways, Gallant and Frost are examples of this new type of hero: the ‘sensitive and brave subject who reads the signs and boldly decides on how to intervene on the spot’ (Krasmann and Hentschel 2019, 192). However, as the following section illustrates, this does not mean that both were seen as heroes. As I hope to show, a distinguishing feature of situational heroism is that it does not require heroes (in the traditional sense).

## Situational awareness and new modes of heroism

Mobile phone footage of the violent struggle on London Bridge went viral within hours. Soon, Frost was known around the globe as the ‘Narwhal Tusk’ hero. A hero is widely understood as a ‘person who knowingly, and voluntarily, acts for the good of one or more people at significant risk to the self, without being motivated by reward’ (Kohen, Langdon, and Riches 2019, 619). This is exactly what Frost did. Yet, he felt uneasy about being called a hero. As Bröckling (2020) highlights, a reluctance to embrace the hero label can itself be seen as a heroic thing to do. In Frost’s case, there were at least two reasons why he felt uncomfortable about being called a hero. A first reason is that he felt that the hero label glosses over the traumatic nature of his experience:

There was also some interesting times when I had other friends telling me that they always wanted to be a hero and they love that idea of it, and it just seems so odd to me because there’s this ideal of a hero and what it is and all the rest, but to do such an act of heroism, there’s a lot of negative consequences that nobody seems to know, or – the wider picture. Like everyone just sees that incident. But that incident on the bridge, strangely enough, it looks quite … it’s crazy. What happened was a crazy thing and the timing and everything I can go into detail, but it’s almost like a miracle what happened in that moment. But there was a lot of horrors that we saw in Fishmongers’ Hall before, and things that I can’t shake from my mind and a lot of, uh, mental health issues that, uh, have come out as a result of that. And, uh, I’m trying to get my brain to make sense of things and to stop playing things over and over and over. There are a lot of negative consequences.

It’s not all just fluffy and celebrating heroism and then walking around like, like a champion. And in fact, I’ve done quite the opposite. I have become a bit of a hermit, I’ve hidden away from people and I used to be very social, whereas now, I kind of shun social environments

(Darryn Frost in zoom interview with author on 16 July 2020)

Like many other survivors of violent attacks, Frost continued to feel the affective force of the situation long after the event. In 2020, he said: ‘it has been horrific for me in trying to overcome, um, the trauma, because I can see it so visually and vividly’. When I spoke to him again three years later, Frost had discussed his experience in numerous media interviews and had become an important advocate for prisoner rehabilitation. Yet he continues to feel the emotional impact of the attack, and there are still some sensory triggers that can throw him back into the situation. Such triggers include loud shouts and sirens, as well as news coverage of world events. In October 2023, he said: ‘whenever I see hurt, death, destruction, anything. There was a terror attack in Brussels yesterday with victims, and so those things bring it all back again’. Frost’s testimony illustrates that even individuals with an exceptional ability to ‘switch into hero mode’ in a violent situation, can find it difficult (if not impossible) to get to the point where they feel that this violent situation is well and truly over.

The second reason that Frost felt uncomfortable with the hero label is that he noticed that it was applied very selectively. In July 2020, he told me:

Um, so yeah, so that was really difficult for me as well, seeing how other people were not treated the same as me, even though they had saved as many lives if not more, and had been as heroic, if not more, and Steve Gallant was on his, like, a day release and he … he took massive risks to, to help other people. He ran against, I think it might have even been against the instructions of his prison officer who was telling him to stay still, but he went, ran straight into danger, regardless. And that could have affected his life and his, and his sentence and all the rest. But he still chose to put other people first and I think that is *so honourable*.

Against this background, Frost chose to embrace the hero label for strategic reasons to campaign for Steve Gallant and other prisoners who had tackled Khan with him. Steve Gallant was a convicted murderer. In 2005, he had received a life sentence for killing former firefighter Barrie Jackson outside a pub in Hull. He was nearing the end of his prison sentence for this crime when he was granted permission to attend the Learning Together event in London. When Gallant’s name was first mentioned in news reports, this had profound consequences for the family of his victim. Jackson’s former partner Vicky Foster said that the impact on her family of the news coverage was ‘devastating’: ‘It re-triggered old trauma… There were flashbacks, nightmares, that lasted months’ (BBC 2020). Elsewhere, she explained, ‘Gallant no doubt helped save lives on London Bridge last year, but he has also caused lifelong damage to myself and my family’ (Foster 2020). While Gallant may not be a hero in the conventional sense, he has clearly acted heroically on 29 November 2019. This, one could argue, makes him a situational hero.

It is no coincidence that the rise of situational awareness went hand in hand with a shift in the understanding of heroism. Building on the findings of the controversial Stanford prison experiment, Philip G. Zimbardo and his team now focus on the ‘banality of heroism’. They insist that we should stop thinking of heroes as ‘superhumans’ with ‘rare personal characteristics’ (Franco and Zimbardo 2006). Instead, they suggest, heroism should be conceived of as a ‘universal attribute of human nature’. According to them, ‘the situation and the personal characteristics of each person caught up in the situation interact in unique ways’. This approach to heroism shifts the focus from heroes to heroic acts and from individual character traits to situational awareness.

Gallant and Frost’s testimonies suggest that they did not identify as heroes. Rather, they portray themselves as people who took action to protect others and had to learn to act in this way. Frost told me that he had to train himself ‘to stand up to people who are attacking others’ while growing up in South Africa. After witnessing the structural violence of the Apartheid regime and bullying in school, he taught himself to ‘override’ his ‘fear factor when someone else is in need’. He was open about the fact that this learned behaviour got him in trouble on some occasions and added jokingly: ‘my girlfriend thinks that I have got a death wish’. The situational hero must perform a delicate balancing act: he or she needs to take quick and bold decisions whilst carefully assessing potential risks and avoiding excessive violence. Gallant was clearly not always able to act in this manner, but his testimony suggests that he taught himself to ‘act like a hero’. On 21 June 2021, he made his case for early release to a parole board. When asked about potential similarities between his attack on Barrie Jackson and his attack on Usman Khan, he explained that the former ‘was motivated by a now defunct set of cognitive deficiencies layered over an unhealthy belief about violence, while London Bridge was more instinctive and selfless’ (Gallant 2023, 284). On 3 August 2021, he was released.

On 17 March 2023, the British Cabinet Office published the last Civilian Gallantry List to be approved by the late Queen Elizabeth II. Fifteen people were honoured for putting ‘themselves at risk to save (or attempt to save) another person’s life’ (Cabinet Office 2023). Eight people were awarded the Queen’s Commendation for Bravery, and seven received the Queen’s Gallantry Medal ‘for acts of exemplary bravery’ (Honours and Appointments Secretariat 2023). Awards are not necessarily given to individuals who faced the greatest risk (although that is one factor in the decision). A key criterion in the selection process is ‘how aware the nominee was of the danger’ of the situation in which they found themselves (Honours and Appointments Secretariat 2023). Being awarded a Queen’s Gallantry Medal meant a lot to Frost, especially because Steve Gallant, Łukasz Koczocik and John Crilly received the same honour. Frost told me with pride that this was the first time that a group of immigrants and prisoners had received this award.

## Conclusion

As this article has shown, violent situations co-exist and intersect, and they can connect people across time and space in life-changing and unexpected ways. The violent situation is ‘a multiplicity that preexists one’s participation in it’ (Zigon 2015, 503). As a result, violent situations do not necessarily have a clearly defined beginning, and they certainly do not have a clearly defined end. While individual testimonies can never fully capture or represent the affective force of violent situations, this article has shown that such accounts can offer critical insights into the ways in which different people sense danger and make sense of their experience. Individual narratives evolve over time in response to a range of internal and external processes (e.g. therapy, news coverage, etc). (Re)narrating how they have experienced violent situations can give individuals a sense of agency and control. However, while testimonies can create relations between different violent situations (e.g. in the past and present), they can never contain them. Every testimony can be challenged.

This article has offered a close reading of testimonies by two survivors of the London Bridge attack in 2019. Steve Gallant and Darryn Frost were spurred into action by the same events. The analysis showed that there are important similarities and overlaps in their testimonies. Yet, it also revealed that the two accounts are characterised by different sensory markers. Gallant’s testimony places great emphasis on sound, whereas Frost’s is dominated by visual sensory markers. There were also significant silences and absences. Although Frost’s memory of the situation was ‘crystal clear’, he found it impossible and/or inappropriate to verbalise some of the things he saw. Of course, it is also important to consider which testimonies are missing altogether: Jack Merritt and Saskia Jones were brutally killed and are no longer able to tell their stories. Łukasz Koczocik, Stephanie Szczotko and other survivors of the attack gave evidence at the Fishmongers’ Hall inquests in 2021 but kept a low profile and did not discuss their experiences in publicly outside this context. Many others whose lives were impacted by the attack made the same choice.

As this article has shown, a close reading of testimonies cannot ignore their ‘performative force’ (Krämer and Weigel 2017). Frost was quickly identified, and he was celebrated in the media as the ‘narwhal tusk’ hero – even if he did not feel very comfortable with this label. Gallant, by contrast, was prevented from sharing his testimony with the public until much later. Gallant was no hero in the conventional sense. As a convicted murderer who was serving a life sentence, he was not granted the same ‘epistemic agency’ (Çaylı 2021, 485) as Frost. Against this background, it can be seen as a remarkable development that he, too, received a Queen’s Gallantry Medal for acts of exemplary bravery.

The social shift towards situational awareness in the UK creates the possibility of new modes of heroism. In the face of a violent situation, Frost and Gallant felt an urge to *do something* to protect others. They had no training or expert knowledge. Instead, they relied on their experience and intuition to decide what to do. Their bold and brave actions on 29 November 2019 exemplify what I call situational heroism. The London Bridge Attack 2019 illustrates the democratic potential of this new form heroism: ordinary citizens and even former criminal can act heroically in a particular situation and be celebrated for their heroic actions. However, this reconceptualisation of heroism raises ethical questions. Vicky Foster had to raise her children without a father because Gallant killed him. How does the violent situation in 2019 relate to the violent situation resulting in Barrie Jackson’s death? Can and should discussions about heroism focus on one particular situation? Foster’s view is clear: ‘Turning your life around is an amazingly brave, heroic thing to do, and if Steve Gallant has done that, then he deserves respect for it. But five minutes on London Bridge would not be proof’ (Foster 2020).

There also exists another ethical question: what happens if situational heroism becomes a moral imperative? In recent years, the UK and other Western nations have seen a wave of politically motivated attacks against ‘soft targets’and public spaces. Many of these attacks were carried out by lone-actor terrorists, who are ‘generally considered to operate outside of a command structured and decide individually when to attack’ (Kenyon, Baker-Beall, and Binder 2023, 2054). The notion that ‘lone actors’, often described as ‘lone wolves’, ‘plan and perpetrate acts of terrorism all by themselves’has been the subject of extensive criticism (Berntzen and Bjørgo 2021, 133; see also McCulloch et al. 2019). Despite this criticism, lone-actor terrorism has become a major security concern and a focus of counterterrorism efforts in many countries. Particularly attacks that are carried out with everyday objects such as knives or vehicles tend to be difficult to detect in the planning stage (Miller and Hayward 2019). Against this background, the public praise for the ‘heroic actions’ (Cabinet Office 2023) of civilians with a high degree of situational awareness has great social and political significance: they are the living proof that it is possible to stop such terror attacks. To ensure that other people can do the same, the National Counterterrorism Security Office and other official bodies supporting the ‘Protect’ and ‘Prepare’ strands of the UK’s counterterrorism strategy CONTEST release regular guidance. A key element of the prepare strategy is to increase individual and organisational awareness because this is considered a vital step in reducing ‘the likelihood of a successful terrorist attack’ (Protect UK 2023). Citizens are encouraged to ‘stay alert’ and to trust their ‘instincts’. What this means in practice, however, is as open as the situation itself.

## Notes

1. There were two periods of significant media interest in the London Bridge attack 2019: the first began immediately after the attack and lasted for a few weeks. Public interest in the case reached another peak during inquests of the victims and perpetrator in 2021. The first book dealing with the attack was published in 2022 by Preti Taneja, who taught creative writing to Usman Khan and other prisoners as part of the Learning Together Programme. Taneja’s novella ‘Aftermath’ (Taneja 2022) focuses on the traumatic impact of the attack in 2019 and explores the intricate relationship between terror, language, and justice. Steve Gallant was the first (and so far, the only) survivor who published an auto-biographical account of the attack. In August 2023, Channel 4 aired the documentary ‘London Bridge: Facing Terror’ featuring interviews with Darryn Frost, Steve Gallant, and John Crilly.

2. Initially, scholarship on Holocaust testimonies focused on auto-biographical accounts of survivors and trials against leading National Socialists. Today it covers a broad range of media and art forms. Recent publications in English include Dean 2019 (de Jong 2018; Hughes 2018; Matthäus and Roseman 2009; Pollin-Galay 2018; Shenker 2015).,

3. The semi-structured interview with Frost was carried out via zoom. It consisted of a 90-minute conversation with the author on 16 July 2020, and a 45-minute follow-up interview on 17 October 2023.

4. Frost correctly identified two severely injured women in that part of the building. However, one survived the attack and was not included in the list of victims. This was confusing for Frost. In 2020, he told me: ‘then, when, uh, the media reports came out that it was Jack and Saskia that died, I started double-guessing myself, or, second-guessing, and thinking how could I have got that wrong? But I must have got that wrong because they were so severely injured. They couldn’t have survived. And so then in my second report, a week later on the next Friday. A week later I had to revisit my, um, statements and go through the transcript. And I had, from media reports, I second-guessed myself and stuff. I must have been wrong of what I was seeing, it doesn’t seem like … And so rather than saying *she* was laying there, I changed my statement to *they* were lying there because that doubt had penetrated my mind’.
